# Type I regulatory T cells in malaria: of mice and men

**DOI:** 10.1172/JCI166019

**Published:** 2023-01-03

**Authors:** Jason Nideffer, Prasanna Jagannathan

**Affiliations:** Department of Medicine, Stanford University School of Medicine, Stanford, California, USA.

## Abstract

Type I regulatory T (Tr1) cells are a population of regulatory CD4^+^ T cells implicated in the suppression of pathological immune responses across multiple diseases, but a unifying transcriptional signature of Tr1 identity across disease contexts has not been characterized. In this issue of the *JCI*, Edward, Ng, and colleagues identified a conserved transcriptional signature that distinguished Tr1 (IL-10^+^IFN-γ^+^) from Th1 (IL-10^–^IFN-γ^+^) cells in human and mouse malaria. This signature implicated genes encoding inhibitory receptors — including CTLA-4 and LAG-3 — and transcription factors — including cMAF. The authors identified coinhibitory receptor expression that distinguished Tr1 cells from other CD4^+^ T cell subsets. Furthermore, cMAF — and, to a lesser extent, BLIMP-1 — promoted IL-10 production in human CD4^+^ T cells. BLIMP-1 also played a role in supporting the expression of inhibitory receptors. These findings describe a few key features that seem to be conserved by Tr1 cells across multiple species, disease contexts, and marker definitions.

## Defining Tr1 cells

The immune system has evolved a variety of mechanisms to regulate immune functions and ensure that inflammatory responses do not escalate far beyond that which benefits the host. Type I regulatory T (Tr1) cells, a subset of CD4^+^ T cells, are thought to play an important role in tempering the immune response by suppressing the inflammatory programs of myeloid cells and other T cells (1). Tr1 cells are considered a regulatory subset of T cells but differ from conventional Tregs in that they do not constitutively express FOXP3 (1). Tr1 cells have been implicated in the outcomes of a variety of clinically relevant diseases. They were found to be relatively abundant in patients with SCID that received hematopoietic stem cell transplantation and exhibited tolerance of the allograft (2). These Tr1 cells were primarily of donor origin and mitigated graft-versus-host disease (GvHD) by inducing antigen-specific tolerance of host HLA alleles. Since their discovery, Tr1 cells have been identified in the context of colitis (3), diabetes (4), bee venom allergy (5), dengue (6), and malaria (7, 8). Notably, Tr1 cells increase in frequency as children living in malaria-endemic settings are repeatedly exposed to the causal parasite, *Plasmodium falciparum* (7). T cell production of IL-10, a hallmark Tr1 cytokine, has also been associated with decreased disease severity in similar cohorts of children (9). Additionally, mice with a T cell-targeted IL-10 deficiency are susceptible to greater disease severity following *Plasmodium* infection and display increased weight loss, anemia, hypothermia, and death compared with WT mice (10). Taken together, Tr1 cells develop in a variety of different contexts and play a critical role in regulating immunity and mitigating immune-mediated pathologies.

While Tr1 cells have been studied in a variety of different contexts, there is substantial uncertainty with respect to their heterogeneity across disease settings. Furthermore, the relatedness of human and mouse Tr1 cells is not fully characterized. In this issue of the *JCI*, Edward, Ng, and colleagues tackle Tr1 heterogeneity as it pertains to disease setting and host organism (11).

## Tr1 cells of mice and men

Edward, Ng, and colleagues defined Tr1 cells as CD4^+^ T cells coexpressing IFN-γ and IL-10, while Th1 cells were defined as CD4^+^ T cells expressing IFN-γ but not IL-10. These definitions were used to sort cells for comparative transcriptomics experiments in humans voluntarily challenged with malaria and in an experimental mouse model. Edward and Ng, et al. reported 1,315 genes that were differentially expressed between human Tr1 and Th1 cells; 159 of these genes were consistent in their differential expression between mouse Tr1 and Th1 cells (11). This interspecies Tr1 signature included the upregulation of the gene encoding cMAF, a positive regulator of IL-10 expression thought to be important for Tr1 function (12). Additionally, BLIMP-1 was predicted, based on pathway analysis, to be more active in human Tr1 cells than human Th1 cells, and the ortholog of this gene was upregulated in mouse Tr1 cells. Similar to cMAF, BLIMP-1 was previously shown to promote IL-10 expression (13, 14); therefore, it makes sense that the activity of these transcription factors would distinguish a population of T cells defined by their expression of IL-10.

In addition to transcription factors previously implicated in the regulation of Tr1 function, coinhibitory receptor genes such as *CTLA4*, *HAVCR2*, and *LAG3* were also consistently upregulated in human and mouse Tr1 cells (11). These receptors have been previously described on Tr1 cells, and LAG-3 is even used — in combination with CD49b — as a phenotypic marker for this population. The gene encoding chemokine receptor CCR5, which has been used in combination with PD-1 to identify Tr1 cells, was also upregulated in both human and mouse Tr1 cells. These data suggest that human and mouse Tr1 cells bear transcriptional similarities and are likely orthogonal cell populations serving overlapping functions (11).

## Tr1 cells across diseases

Edward, Ng, and colleagues compared their Tr1 signature from people infected with malaria to previously published signatures of Tr1 cells from people infected with dengue virus (6) and Tr1 cells generated — for the purpose of treating GvHD — by ex vivo activation of peripheral blood-derived naive CD4^+^ T cells in the presence of tolerogenic dendritic cells (DC-10) and IL-10 (15). This comparison was, however, limited by the fact that the Tr1 and comparator definitions differed in each of the three studies. The dengue study defines Tr1 cells identically to Edward and Ng, et al., but it compares them with IFN-γ^–^IL-10^–^CD4^+^ T cells instead of IFN-γ^+^IL-10^–^CD4^+^ Th1 cells (6). The GvHD study defines Tr1 cells as LAG-3^+^CD49b^+^CD4^+^ T cells and compares them with LAG-3^‑^CD49b^–^CD4^+^ T cells (15). With these experimental differences in mind, Edward and Ng, et al. identified eight genes that were consistently upregulated by Tr1 cells across the three settings. Included among these genes were *CTLA4*, *HAVCR2*, and *LAG3*, which similarly unified human and mouse Tr1 signatures (11). Despite these similarities, the Tr1 signatures across the three studies were quite distinct, and while this finding may indicate the heterogeneity of Tr1 cells across disease contexts, differences in the Tr1 and comparator definitions may be confounding.

## Tr1 coinhibitory receptors and transcription factors

Given that coinhibitory receptors were common to the Tr1 signatures of mice and humans across multiple disease settings, they are likely a key feature of Tr1 identity. Furthermore, Edward and Ng, et al. report that CTLA-4, PD1, and ICOS increase in abundance on the surface of LAG-3^+^CD49b^+^CD4^+^ T cells following controlled human malaria infection (11). Notably, these cells also upregulated cMAF and BLIMP-1. These findings demonstrate that a coinhibitory receptor-rich population of Tr1 cells increases in frequency following *Plasmodium* infection in humans, supporting previously made associations between prior parasitemia and Tr1 abundance.

The authors demonstrated that human LAG-3^+^CD49b^+^CD4^+^ T cells expressed IL-10 to a greater extent than other CD4^+^ T cell populations in the context of *Plasmodium* infection. However, the degree to which the LAG-3^+^CD49b^+^ and IFN-γ^+^IL-10^+^ populations overlap was not directly assessed, leaving open questions about whether these Tr1 definitions demarcate the same population. From their mouse studies, the authors demonstrated only partial overlap between these populations, with not all LAG-3^+^CD49b^+^CD4^+^ T cells producing IL-10 and a subpopulation of LAG-3^–^CD49b^–^ CD4^+^ T cells seemingly producing the most IL-10. In the infected mice, IL-10 and IFN-γ expression correlated more strongly with the expression of CCR5 and TIGIT than with CD49b or LAG-3 (11).

BLIMP-1 and cMAF were transcription factors upregulated in both human and mouse Tr1 cells. Edward, Ng, and colleagues knocked down the expression of these factors in human CD4^+^ T cells using CRISPR/Cas9 and assessed their affect on *in vitro* differentiation. They demonstrated that both BLIMP-1 and cMAF from CD4^+^ T cells were important for IL-10 production, while BLIMP-1 played an additional role in promoting the expression of coinhibitory receptors (11). These results recapitulate those of a recently published study that identified BLIMP-1 and cMAF as components of a molecular switch that induces IL-10 and coinhibitory receptor expression in CD4^+^ T cells (16). In that study, decreased IL-10 production coincided with lower levels of both BLIMP-1 and cMAF in memory CD4^+^ T cells of patients with Crohn’s disease (16). The work of Edward and Ng, et al. ultimately supports prior studies implicating BLIMP-1 and cMAF as crucial molecular regulators of Tr1 cell function and highlights the utility of CRISPR-based methods for studying the differentiation and functions of memory CD4^+^ T cell subsets (11).

## Redefining Tr1 cells

**Figure 1:**
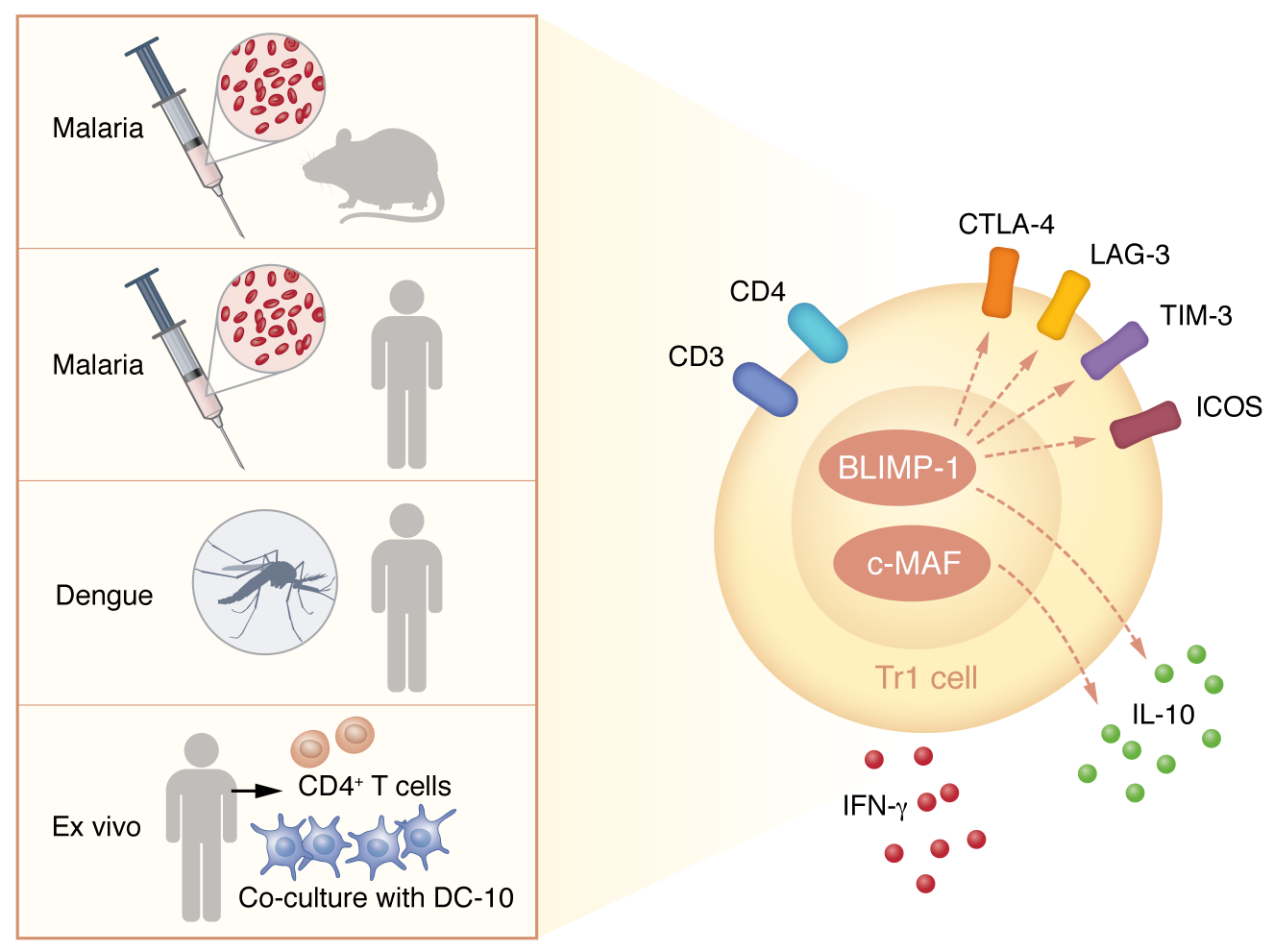
Select features of Tr1 biology are conserved across disease models. Tr1 cells from four different contexts, including mouse malaria, human malaria, human dengue infection, and human CD4^+^ T cells cultured ex vivo under differentiating conditions in the presence of activating tolerogenic DC-10 and IL-10, share a common phenotype. This interspecies Tr1 signature is characterized by the expression of coinhibitory and stimulatory receptors (CTLA-4, LAG-3, TIM-3, ICOS) and by the production of IL-10 and IFN-γ. In ex vivo Tr1 differentiation experiments, BLIMP-1 and cMAF promoted the expression of IL-10, and BLIMP-1 played an additional role in positively regulating coinhibitory receptor expression.

There is hardly a consensus on the markers that identify Tr1 cells — various publications define them as CD4^+^ T cells that express IL-10 and IFN-γ (1), CD49b and LAG-3 (1), or CCR5 and PD-1 (17). The lack of an agreed upon Tr1 phenotype may, in part, stem from the assumption that any suppressive CD4^+^ T cell that doesn’t express FOXP3 is a Tr1 cell. Thus, whether these various marker combinations define the same Tr1 population or whether they represent distinct regulatory subsets remains uncertain. In light of Edward and Ng, et al., we suspect that the Tr1 designation is currently being used to define diverse cell populations that suppress immune responses through various mechanisms. However, there are certain aspects of Tr1 biology that appear to be conserved across disease models, such as the expression of IL-10 and specific coinhibitory receptors largely regulated by c-MAF and BLIMP-1, respectively (Figure 1). This knowledge will aid in the identification of Tr1 signatures that correspond with a variety of disease contexts, but certainly, more research is needed to completely answer the question: what are Tr1 cells?

Future efforts to characterize Tr1 heterogeneity and ontogeny should consider unbiased, single-cell assessments of memory CD4^+^ clonal, transcriptomic, and epigenetic diversity, ideally without prior cell sorting/selection based on surface-marker expression. Such an approach could leverage unsupervised clustering to determine whether there is a population of cells bearing a Tr1 signature that is truly distinct from other memory CD4^+^ T cell subsets. This approach could also tease apart Tr1 cell heterogeneity. Furthermore, single-cell data of this nature would be comparable across studies of various diseases without concern for the confounding effects of inconsistent Tr1 cell and comparator subset definitions. Enriching for T cell receptor transcripts prior to sequencing would also allow for the determination of clonal relationships between Tr1 cells and other memory CD4^+^ T cell subsets and would provide insight into their ontogeny. Most importantly, in order to know for certain what Tr1 cells are, it will be critical to determine how the heterogeneity of transcriptional and epigenetic programs of Tr1 cells contribute to their functional capabilities.

## Version 1 01/03/2023

Electronic publication
